# Annotations

**Published:** 1893-11-18

**Authors:** 


### ANNOTATIONS.

### Malthus Redivivus.

THE spread of sociological principles having a physiological basis is well illustrated by the unexpected terms in which an audience of artisans, consisting of close upon two thousand persons, were addressed by the Rev. Dr. R. F. Horton a few Sunday evenings ago. Dr. Horton sternly declared that it was nothing but "gross selfishness" to bring children into the world where there is no probability of maintaining them decently. Too often, he said, not only were helpless infants born to dirt, disease, and misery, but to almost inevitable moral and spiritual ruin. Dr. Horton sees the moral and spiritual aspects of the question; the physiologist sees also the physiological. It can hardly be denied that population in Great Britain is increasing out of all proportion to our resources. The inevitable results of misery and physical deterioration must and do follow. The really important question is, whether anything of a practical nature can be done; and, if so, what? There are two courses open—we may either persuade individuals one by one, or we may enact laws. The persuasion method might possibly produce useful results in the course of two or three thousand years if it were intelligently and systematically carried out, though the experience of the world during the period between Aristotle and Malthus is not encouraging. For our part we have more faith in the immediate effect of law and penalty. The educated man makes his own law on this question, and abides by it. It is the rule with him, a rule which has few exceptions, to delay marriage until his resources justify the step. The mass of the uneducated, on the other hand, rush into marriage with no more regard for the future than the wild animals of the woods. But we should put legal checks upon them. If marriages were delayed by statute among the working class until the ages of twenty-seven for men and twenty-five for women, an astonishing result would be apparent even in a single decade. The effects would be a marked diminution in the rate of increase among the population, and a correspondingly marked improvement of general physique. As things are at present the population increases in numbers with extraordinary rapidity, and as rapidly goes down hill in physique and morale. This means certain ruin to the whole community if it be not checked.

### A B C Diet.

THERE is an appearance of charming simplicity about anything which can be reduced like the well-known Railway Guide, to the formula expressed by A B C. But a whisper has been heard in the City that A B C, in so far as diet is concerned, has been tried and found wanting. In the olden days of ten or twelve years ago a chop or a steak with potatoes, followed by bread and cheese and a glass of beer or stout, was considered the orthodox thing. Strong men, and capable fiancial thinkers were nourished on this homely but robust English dietary. Nowadays the chop, the steak, the beer, and the cheese are alike abandoned. Instead thereof, some bread and butter, followed by a "cake," with a cup of coffee or tea, constitutes the midday replenishment of physiological energy. Occasionally, perhaps, a thin slice of cold beef, or ham, or an egg, is added to such Lenten fare. This A B C method has been in progress for a considerable number of years, and we can now look upon its results on an extensive scale. "Anæmia" is whispered in the city; anæmia, not of women, but of men—bloodlessness, in plain English. The city clerk of the period, nourished on a midday diet of A B C, is said to be a bloodless animal, incapable of either hard work or protracted endurance. If this be true it is a serious business. When one remembers that with many young men of the clerk class, perhaps with most, the midday meal, politely called "lunch," is really "dinner," one feels a distinct accession of anxiety on behalf of both them and their children. In point of fact, a bread-and-butter and coffee luncheon, which is really the dinner of the day, is a very serious matter for young men. It means, for certain, both bloodlessness and physiological exhaustion; it means, moreover, incapacity for business and premature old age; and, in the last resort, it means the degeneration and extinction of descendants. In our climate, and most decidedly for those whose lives are spent in offices, some meat is essential. Where a good all-round dinner is not taken in the evening, a solid meat meal should be eaten in the middle of the day. This requires no argumentation. City clerks, whose mirrors tell them of pale faces, and whose feebleness is in marked contrast with the energy of their country and schoolboy days, must look to their midday meals. If they do not, their stomachs, defrauded of the necessary pabulum, will sooner or later call in the policemen of bloodlessness, incapacity, chronic illness, and shortened days.

### Progress in Port Sanitation.

DR. COLLINGRIDGE's half-yearly Report on the sanitary work and condition of the Port of London is of more than usual interest in view of the continued existence and spread of the cholera epidemic on the Continent and in various trade centres connected by regular business communication with our own metropolis. The Report is remarkable for two things. In the first place, Dr. Collingridge informs us, and there is no higher authority on the subject, that the foreshores and the waters of the Thames are beginning to show decided progress .towards wholesomeness as the result of the sanitary energy of the London County Council. He anticipates, as we do, that in no long time we shall have a river, not only of beauty, but of quite remarkable purity. The other and most urgent part of Dr. Collingridge's report deals with the necessity for the medical inspection of all ships and their crews which crowd the waters of the Thames. "Medical inspection," and nothing but medical inspection, "constant and continuous," can give us adequate guarantees of sanitary security in such a port as this of London. Ordinary people have no doubt assumed that such "constant and continuous medical inspection" of ships and their crews entering all our larger ports has been the rule for many years past. It ought to have been, of course; but, as a matter of fact, it was not until the summer just ended that, frightened by the near approach of cholera, we consented to be at the expense of proper medical inspection of ships and crews. The medical officer tells us that during the six months just past 8,773 ships entering the port were inspected, and that of these so many as 341 required special orders as to cleansing. In other words, there were 341 vessels during the six months, or about two every day, which might have spread dangerous diseases throughout London and the country but for the competent inspection of the medical officers of the port. The actual number of individuals inspected in the various ships was no less than 42,307. The possibilities of danger from such a number of very mixed persons were enormous; whilst competent inspection was all that was required to nip almost every variety of dangerous infectious disease in the bud. We heartily endorse Dr. Collingridge's recommendation that the special inspectorships established for and during the cholera alarm shall be permanently continued as part of our normal resources for the sanitation and safety of the Port of London.

